# Integrating Network Pharmacology and Metabolomics to Elucidate the Mechanism of Action of Huang Qin Decoction for Treament of Diabetic Liver Injury

**DOI:** 10.3389/fphar.2022.899043

**Published:** 2022-05-25

**Authors:** Xiaomin Xu, Cheng Fang, Yu Wang, Fang Lu, Shumin Liu

**Affiliations:** ^1^ Research Institute of Traditional Chinese Medicine, Heilongjiang University of Traditional Chinese Medicine, Harbin, China; ^2^ Drug Safety Evaluation Center of Heilongjiang University of Chinese Medicine, China

**Keywords:** metabolomics, network pharmacology, huang qin decoction, diabetic liver injury, metabolite

## Abstract

Huang Qin Decoction (HQD), is used for the treatment of diabetic liver injury (DLI) and in this study, its mechanisms were evaluated by metabonomics and system pharmacology. To study the anti-DLI effects of HQD. The 48 male db/db mice were fed adaptively for one week, and a random blood glucose test was performed twice. The db/db mice with a blood glucose level of more than 11.1mol/l were separated into four groups: the model group, the active control group, the high-dose HQD group the low-dose HQD group, the control group consisted of db/m mice. Using the UHPLC/Q-TOF-MS metabolomics approach, 18 metabolites were found to be profoundly altered in the model group, and the levels of these biomarkers were significantly recovered after treatment with HQD. 8 signaling pathways related to HQD, including the Sphingolipid metabolism, Taurine and hypotaurine metabolism, Phenylalanine metabolism, Glutathione metabolism and Glycerophospholipid metabolism, etc. were explored. In addition, the system pharmacology paradigm revealed that HQD contains 141 active ingredients and is related to 265 genes, and 1404 disease genes are related to DLI. The construction of the HQD composition-target-DLI network identified a total of 161 intersection genes. We identified 10 key genes, which is partially compatible with the results of metabolomics. The integrated approach metabolomics and network pharmacology revealed that additional detailed investigation focused on five major targets, including CAT, PTGS2, MAPK3, AKT1, and MAPK8, and their essential metabolites (sphinganine, sphingosine, Glutahione, Oxidized gutahione, Dihydrolipoamide) and pathway (glycerol phospholipid metabolism and tryptophan metabolism). The significant affinity of the primary target for the HQD was confirmed by molecular docking. The results demonstrate that the combination of metabolomics and network pharmacology could be used to reflect the effects of HQD on the biological network and metabolic state of DLI and to evaluate the drug efficacy of HQD and its related mechanisms.

## Introduction

Diabetes is a common metabolic disease characterized by elevated blood sugar levels that may affect nerves, blood vessels, the heart, kidneys, eye, foot, and other organs and tissues, leading to a series of dysfunction and chronic injury. The liver is the main of human metabolism and one of the primary target organs implicated in chronic diseases of diabetes mellitus (Yue et al., 2022). Early liver injury induced by diabetes is sometimes difficult to determine due to the liver’s high compensatory function. At the same time, the liver plays a key role in blood glucose management and sugar storage and distribution. As a result, protecting the liver is as important as controlling blood sugar in the management of diabetes (Tanase et al., 2020).

DLI results from multiple factors and mechanisms, such as damage, anti-injury, proliferation repair, and comprehensive regulation. Diabetes-complicated liver damage is characterized by inflammation and oxidative stress induced by blood sugar, fat, protein, water, and electrolyte abnormalities, which leads to impaired liver function and glucose and lipid metabolism (Luo et al., 2018; Zhang et al., 2018). Its pathophysiology is complicated, and there is no particular medication therapy for DLI at the moment (Liu et al., 2013). At present, chemical hypoglycemic medicines are frequently used to treat diabetes by oral administration. These treatments have several problems, including considerable toxic and side effects, various adverse responses, a single target, minor effects, and even liver injury. However, traditional chinese medicine compounds have the characteristics of multi-component, multi-target, multi-pathway comprehensive regulation, and comprehensive intervention, which provides a novel approach for the research and prevention of this disease. As a result, we investigate the protective mechanism of the traditional chinese medicine “HQD” against DLI.

HQD is a traditional prescription for removing heat, alleviating diarrhea, and relieving pain from Zhang Zhongjing’s Treatise on Febrile Diseases. It is prepared using the decoction of four traditional Chinese medicines: scutellaria baicalensis (Scutellaria baicalensis Georgi (Lamiaceae) is a popular medicinal plant, Scutellaria baicalensis Georgi), Radix Paeoniae (This product is the dried root of Paeonia lactiflora Pall. Ranunculaceae, Paeonia lactiflora Pall), jujube (This product is the dried and mature fruit of D. Ziziphus jujuba Mill, Ziziphus jujuba Mill), and licorice (This product is the dried root and rhizome of Glycyrrhiza glabra L. legaceae, Glycyrrhiza glabra L). Among them, Scutellaria baicalensis is bitter cold and strong in yin and clears heat in the interior; peony has a slightly bitter and sour taste, relieves pain, restrains yin; bitter and acid complement each other, and has the effect of regulating the middle and preserving yin to stop dysentery. Jujube restores qi and boosts vigor. Data show that HQD has been used in the treatment of gastrointestinal diseases for thousands of years (Enhui C, 2021). Meanwhile, According to modern pharmacological research, HQD contains anti-inflammatory, antibacterial, analgesic, antipyretic, and sedative properties. Scutellaria baicalensis and Radix Paeoniae as the main drugs of decoction, the study has shown participation in the treatment of diabetes and its complications (Cao Min X J et al., 2019; Zheng and Yankun, 2021; Yvette, 2020; Zhang et al., 2021; Fan et al., 2020; Ding et al., 2019; Yan and Jin, 2018; Sun et al., 2019; Gu and Chengjuan, 2020; Radica, 2020; xia et al., 2015), However, whether HQD can treat diabetic liver injury has not been reported, and this experiment elucidate the effect of HQD on diabetic liver injury. Related research has discovered that baicalin, an active ingredient in Scutellaria baicalensis Georgi, has a therapeutic effect on various diabetic complications and can effectively improve rats with diabetes and non-alcoholic fatty liver (Kuo et al., 2019); Paeoniflorin in Radix Paeoniae can inhibit hepatocyte apoptosis by improving oxidative stress and regulating multiple inflammation-related pathways (Wang et al., 2012), Early pharmacodynamic investigations by this study group revealed that HQD may significantly pull back ALT, AST, SOD, and MDA levels, slow down the liver injury, and control blood sugar levels in model mice to achieve preventive and therapeutic disease advantages.

Metabolomics, the qualitative and quantitative analysis of small molecular metabolites in biological samples, as well as the investigation of the relationship between metabolites and research objects, and its application in the discovery of DLI biomarkers are essential for the treatment of a variety of metabolic diseases, including DLI. Biomarkers are important tools for clinical diagnosis. They are regarded as effective methods for integrative research (Wang et al., 2015; Wang et al., 2017). The development of network pharmacology builds on the increasing understanding of protein and molecular interactions and is of great help to understanding the pathogenesis of TCM syndrome and the therapeutic mechanism of TCM (Ning et al., 2018; Guo et al., 2020; Zheng et al., 2021). Therefore, the combination of network pharmacology and metabolomics provides an effective way to scientifically explain the metabolic mechanism of TCM in the treatment of diabetes and its complications.

A metabolomics technique based on ultra-high liquid chromatography-mass spectrometry (UPLC-MS) combined with network pharmacology technology was utilized to demonstrate the therapeutic effect of HQD on DLI to identify potential therapeutic targets. Create a DLI model and use liver metabolomics to test for various metabolites. After that, use network pharmacology to identify possible targets for HQD in the treatment of DLI and to create a complete network of metabolomics and network pharmacology. Finally, molecular docking is used to validate the obtained key targets. This research used a combination of metabolomics and network pharmacology to develop a technique for understanding the potential mechanism of DLI and identifying prospective targets for HQD in DLI management.

## Experimental Materials and Methods

### Experimental Materials

Scutellaria baicalensis Georgi; Radix Paeoniae; Jujube; Licorice (purchased from Hebei Quantai Pharmaceutical Co., Ltd.); Metformin was purchased from Biotopped (CAS NO.: 1115-70-4), Pentobarbital Sodium (SIGMA); Leucine Enkephalin (L9133, Sigma-Aldrich); Methanol (Chromatography grade, Dikma Technology Company); Acetonitrile (Chromatography grade, Dikma Technology Company); Ultra-high liquid chromatography-time-of-flight tandem mass spectrometer (Wates Company, USA); Cryogenic refrigerated centrifuge (Thermo Scientific Company, USA); KQ-500DB Ultrasonic cleaner (Kunshan Ultrasonic Instrument Co., Ltd.); Nichipet EX micro sampler (10–100 μl, 100–1000 μl, NICHIRYO company, Japan); VX-Ⅱ multi-tube vortex oscillator (Beijing Tajin Technology Co., Ltd.); Thermo Scientific 995 ultra-low temperature refrigerator (Thermo Scientific Company, USA); Rotary evaporator (SHZ-III YR1813583).

Preparation of HQD: weigh Radix Scutellariae, Radix Paeoniae Alba, Jujube, and Licorice in a 3:2:2:2 ratio, add 10 times the total weight of water in volume, soak for 30 min, decoct for 1 h, and filter. The filtrate is obtained, and the residue is added to 8 times the volume of water and decocted for 1 h again, filtered to obtain the filtrate, and the filtrate obtained from the two times is mixed and concentrated to 1.5 g/ml. Take out part of the concentrate and dilute it with water according to the ratio of 1:2 to get the final concentration of low-dose HQD of 0.5 g/ml.

Scutellaria baicalensis Georgi; Radix Paeoniae; Jujube; Licorice were acquired from the Hebei Quantai Pharmaceutical Co., Ltd. The voucher specimen of the herb was authenticated by Prof. Zhenyue Wang of the Department of Resources and Development of TCM at Heilongjiang University of Chinese Medicine, and it met the standards of the “Pharmacopoeia of the People’s Republic of China (2020 edition)”. The extract of HQD was obtained from our previous experiments and stored in the theoretical laboratory of TCM properties of Heilongjiang University of Chinese Medicine.

This experimental group has completed the quality standard determination of HQD in the early stage. According to the 2020 Chinese Pharmacopoeia (Committee, 2020), baicalin in Scutellaria should not be less than 8.0%, and paeoniflorin in Radix Paeoniae should not be less than 1.2%. All of these are determined to meet the 2020 Chinese Pharmacopoeia standard. The chromatogram is shown in Fig S.

### Experimental Animals

The 48 male db/db mice and 8 db/m mice were purchased from Airmate Technology Co., Ltd (Animal Certificate Number: No.202009670). The mice were fed in a single cage in a clean barrier system, with free to eat and drink, temperature regulated at 20-26°C, humidity-controlled at 40-70%, and alternating animal lighting in the light and dark cycle maintained 12/12 h. The cages and padding were replaced once a week. All animal experiments were conducted following the relevant regulations of Heilongjiang University of Traditional Chinese Medicine’s experimental animal ethics committee (DWLL20151108001).

The db/db mice were fed adaptively for 1 week, and a random blood glucose test was performed twice. The db/db mice with a blood glucose level of more than 11.1 mol/L were separated into three groups: the model group (3.75 g/kg), active control group (metformin 250 mg/kg): high-dose HQD group (11.25 g/kg), low-dose HQD group (3.75 g/kg), The control group consisted of db/m mice. After grouping, each group was administered according to the 7.5mg/kg administration volume and administered once daily for 8 weeks.

### Sample Processing and Analysis

The mice were anesthetized intraperitoneally with pentobarbital sodium 24 h after the last treatment. Blood samples were taken from the eyeball. After standing (4°C, 60min), the blood samples were centrifuged (3500r/min, 15min, 4°C). After removing the liver tissue, the saline was perfused, sucked dry using filter paper, immediately deposited in liquid nitrogen, frozen, and transported to a - 80°C refrigerator to calculate AST and ATL indices. It is utilized to detect SOD, MDA levels and perform UPLC-TOF-MS analysis. After thawing at room temperature, 9 times the amount of frozen methanol was added for grinding, vortex mixing for 3 min, centrifuged (12000r/min, 15min, 4°C) twice, and the supernatant was kept at - 80°C for SOD and MDA level detection and UHPLC/MS analysis.

### Detection of Random Blood Glucose

Random blood glucose in the mouse tail vein was measured randomly once every two weeks during the experimental cycle.

### Detection of Biochemical Indicators in Biological Tissue Samples

To detect the corresponding indicators, prepare the serum and tissue homogenates and determine the levels of SOD and MDA in liver tissue and the levels of AST and ALT in the serum, according to the kit instructions.

### Liver Histopathological Observation

The left liver lobes of each mice were sliced and immediately placed in a formaldehyde-fixed solution. After 48 h, it was usually preserved in paraffin, sectioned, and stained with hematoxylin and eosin (HE). Under a light microscope, the pathological alterations in liver tissue were examined.

### UPLC-Q-TOF MS Analysis

UPLC conditions: Chromatographic column; ACQUITY UPLC^®^ BEH C_18_ Chromatographic column (2.1 mm × 50 mm, i.d. 1.7 μm), A is acetonitrile (containing 0.05% formic acid), mobile phase B is water (containing 0.05% formic acid), and the gradient elution program is 0–8 min: 98% -60% B; 8–10 min: 60%-2% B; 10–13 min: 2%-0% B; 13–14 min: 0%-98%B; 14–17min: 98%-98%B; flow rate is 0.40 ml/min, injection volume is 2 μl, column temperature is 40°C, sample chamber temperature: 5°C. Mass spectrometry conditions: Electrospray ion source (ESI) utilizes positive and negative ion modes for detection. The locked mass concentration is 2.0 μg L^−1^, and the flow rate is 40 μL min^−1^. Dissolvent gas temperature 350°C, dissolvent gas flow 750.0L/h, ion source temperature 110°C, cone gas flow: 20 L/h, capillary voltage positive ion 1300.0 V, negative ion 1500.0 V; sample cone voltage positive ion 60.0 V, negative ion 70.0 V; use LockSprayTM correction system for online mass correction of leucine enkephalin, data acquisition range m/z 100 ∼ 1500 Da, adopt full scan mode.

### Multivariate Statistical Analysis of Liver Metabolic Profiles and Identification of Potential Biomarkers

Align the raw data processed by UPLC-Q-TOF-MS, then preprocess the peak matching, noise reduction, and normalization data. The data from liver tissue were analyzed using the software ProgenesisQI and EZinfo2.0 (MarkerLynx1.4 workstation software), PCA (principal components analysis) was used for unsupervised statistical analysis, and the samples to be tested were grouped and analyzed using supervised partial least squares discriminant analysis (PLS-DA) to obtain the corresponding score map. SIMCA-P software 12.0 should be used for default cross-validation, and 200 random permutations should be utilized to evaluate the data to prevent overfitting of the PLS-DA model. The S-plot and variable importance projection VIP-plot were used to illustrate the dispersion between groups. The specific metabolites were screened using the score map and the deviation change trend across each ion aggregation point group. The substantially different expressed metabolites were discovered using the same standard. Then, as screening criteria, variable importance projection (VIP) > 1 and *p* < 0.05, fold change (FC) > 1.2 were employed to identify specific prospective biomarkers. Through the human metabolome database (HMDB), the Kyoto Encyclopedia of Genes and Genomes (KEGG), MetaboAnalyst (http://www.metaboanalyst.ca/), and other metabolic pathway databases, enrichment analysis and network construction of related metabolic pathways are conducted, and more information on the physiological and pathological status of differential metabolic markers is provided. A large amount of biological information was collected to search for various metabolites and metabolic pathways connected to disease studies.

### Network Pharmacological Analysis

The active ingredients in HQD were screened using the TCMSP database under the criteria of 30% oral bioavailability (OB) and 18% drug-like activity (DL), and screened components were supplemented with literature reports. The retrieved components are imported successively into the TCMSP database to predict the target and get the relevant target’s protein name. They were imported into the UniProt database for gene name transformation. The corresponding traditional Chinese medicine targets were obtained; the components without relevant targets were screened and removed to obtain the effective HQD components. The Genecards database was utilized to extract “DLI,” and the intersection of disease target and drug target was used as the prediction target of drug effect on disease. The UniProt database has been adopted (http://www.uniprot.org/) to regulate the names of genes and proteins.

The target information of the interaction between HQD and DLI obtained above was imported into the STRING database (https://string-db.org/) to determine the connection between potential targets. The protein-protein interaction analysis results from the string database were visually evaluated using the Cytoscape v3.8.2 software, and a protein-protein interaction network was constructed. GO (gene ontology) annotation and KEGG (Kyoto encyclopedia of genes and genomes) pathway enrichment analysis were performed on the obtained HQD anti-DLI target using the ClueGo + CluePedia plug-in Cytoscape v3.8.2. The pathway with *p* < 0.05 was screened. Metscape is used to import the differential metabolites found in metabolomics into Cytoscape to generate a compound reaction enzyme gene network. This structure is designed to illustrate the interactions between metabolites, pathways, enzymes, and genes. The important metabolites and proteins were then identified by connecting the compound reaction enzyme gene network to the core and metabolic pathways.

### Molecular Docking

The selected key genes were molecularly docked with the core components of HQD and metformin, respectively. Download the 2D structure of the active components in HQD and the positive drug metformin from the PubChem database, save it in “sdf” format, open it using ChemBio3D, optimize the mechanical structure, and save it in “mol2” format. The target protein’s crystal structure is obtained from the RCSB protein database (https://www.rcsb.org/) stored in the pdb format. Convert the ligand and receptor files to pdbqt format using AutoDockTools1.5.6. The structure was improved by eliminating water molecules and replacing them with hydrogen atoms. Autodock Vina was used for the molecular docking investigation.

### Statistical Analysis

Statistical analysis was performed using SPSS (26.0) statistical software by applying Chi Square test. p value less than 0.05 was considered as statistically significant.

## Results

### Random Blood Glucose

**FIGURE 1:**
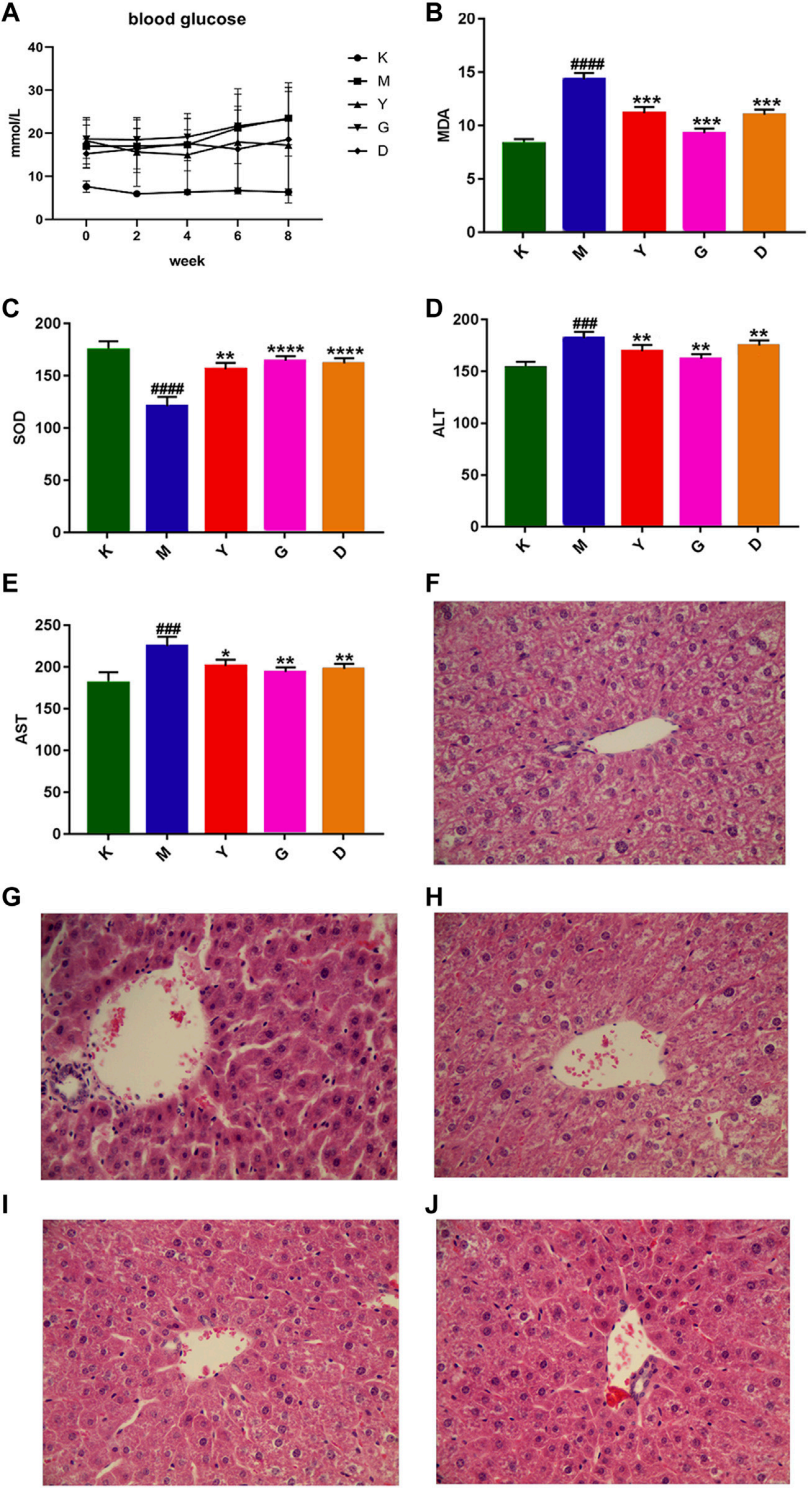
Biochemical indexes were observed together with the histopathological sections. **(A)** Blood glucose changes in mice with DLI (n = 8, ±S); **(B)** Effect of HQD on SOD level in DLI model mice; **(C)** Effect of HQD on MDA level in DLI model mice; **(D)** Effect of HQD on AST level in DLI model mice; **(E)** Effect of HQD on ALT level in DLI model mice; Pathological observation; **(F)** The pathological changes of liver in control group (×20); **(G)** Histopathological changes of liver in model group (×20); **(H)** Histopathological changes of liver in high-dose HQD group (×20); **(I)** Histopathological changes of liver in low-dose HQD group (×20); **(J)** Histopathological changes of liver in active control group (×20). Note: (K: control group; M: model group; Y: Active control group; G: high-dose HQD group; D: low-dose HQD group).

Comparison of mice random blood glucose results found that the control group and the model group was particularly significant (*p* < 0.01). The difference between the positive control group and the model group was significant (*p* < 0.05). However, between the high and low dose groups and the model group (*p* > 0.05), indicating that the high and low doses of HQD had no significant effect on the blood glucose of DLI mice, the detailed results are shown in Figure 1A.

### Observation of the Biochemical Indicators

As seen in Figures 1B–E, serum AST and ALT levels in the model group were significantly higher (*p* < 0.01) than in the control group, indicating that the liver function of mice was damaged. Compared to the model group, the high and low dose HQD group and the active control group could recover serum AST and alt levels, Among them, the high-dose HQD group had the most significant effect. Compared to the control group, the level of SOD in the model group’s liver homogenate decreased significantly (*p* < 0.01), while the level of MDA increased significantly. In the high and low dose HQD group and the active control group, mice’s SOD levels increased while their MDA levels decreased significantly, Among them, the high-dose HQD group had the most significant effect. The results show that HQD reversed blood AST and ALT and SOD and MDA levels in the liver homogenate of DLI mice.

### Histopathological Observation

The liver morphology of mice was studied under the microscope using HE staining. The hepatocytes of mice in the control group were tightly clustered and structurally intact, while the hepatocytes in the model group were enlarged and balloon-like in degeneration. The hepatocytes of mice are tightly grouped after the intervention of HQD and metformin, with the full structure and a small number of vacuoles, Among them, the high-dose HQD group had the most significant effect, as shown in Figures 1F–J.

### Analysis of the Mice Liver Metabolomics Data

**FIGURE 2:**
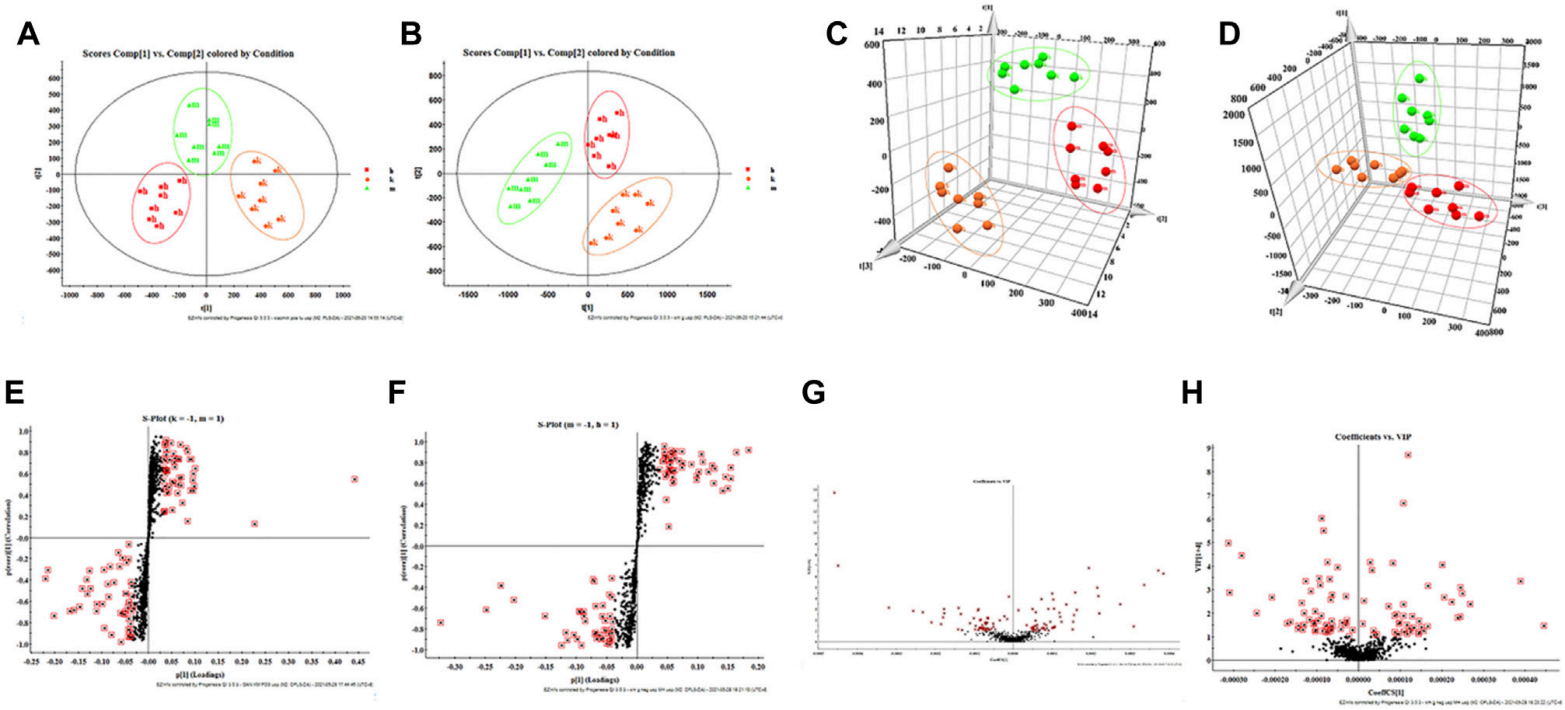
Multivariate statistical analysis of liver metabolism spectrum in DLI mice. **(A)** 2D-PCA score plot in positive ion mode and **(B)** negative ion mode; **(C)** 3D-PCA score plot in positive ion mode and **(D)** negative ion mode; **(E)** S-plot in positive ion mode and **(F)** negative ion mode; **(G)** VIP plot in positive ion mode and **(H)** negative ion mode.

Combining the above pharmacodynamic index analysis, the high dose of HQD was selected for metabolomic analysis. By analyzing the data provided by UPLC-TOF-MS, PCA was processed using unsupervised orthogonal partial least squares discriminant analysis to determine the validity of the metabolic profile of mice liver supernatant samples. The results showed that the control group was significantly separated from the model group, indicating the high reliability of the model established in this study; when comparing the control group, the model group, and the HQD group at the same time, it can be found that each group presents its clustering. The HQD group has the trend of callback to the control group, indicating that HQD can improve the deviation of metabolism in Figure 2A–D.

According to the s-plot, most metabolite ions concentrate near the edges, with just a few ions deviating from it. These ions deviate from the origin, indicating differences between the two groups; by performing the screening requirements of VIP >1, the VIP plot can be obtained, and prospective biomarkers may be preselected, as illustrated in Figure 2E–H.

### Endogenous Potential Biomarkers of Liver Metabolism in Mice

**FIGURE 3:**
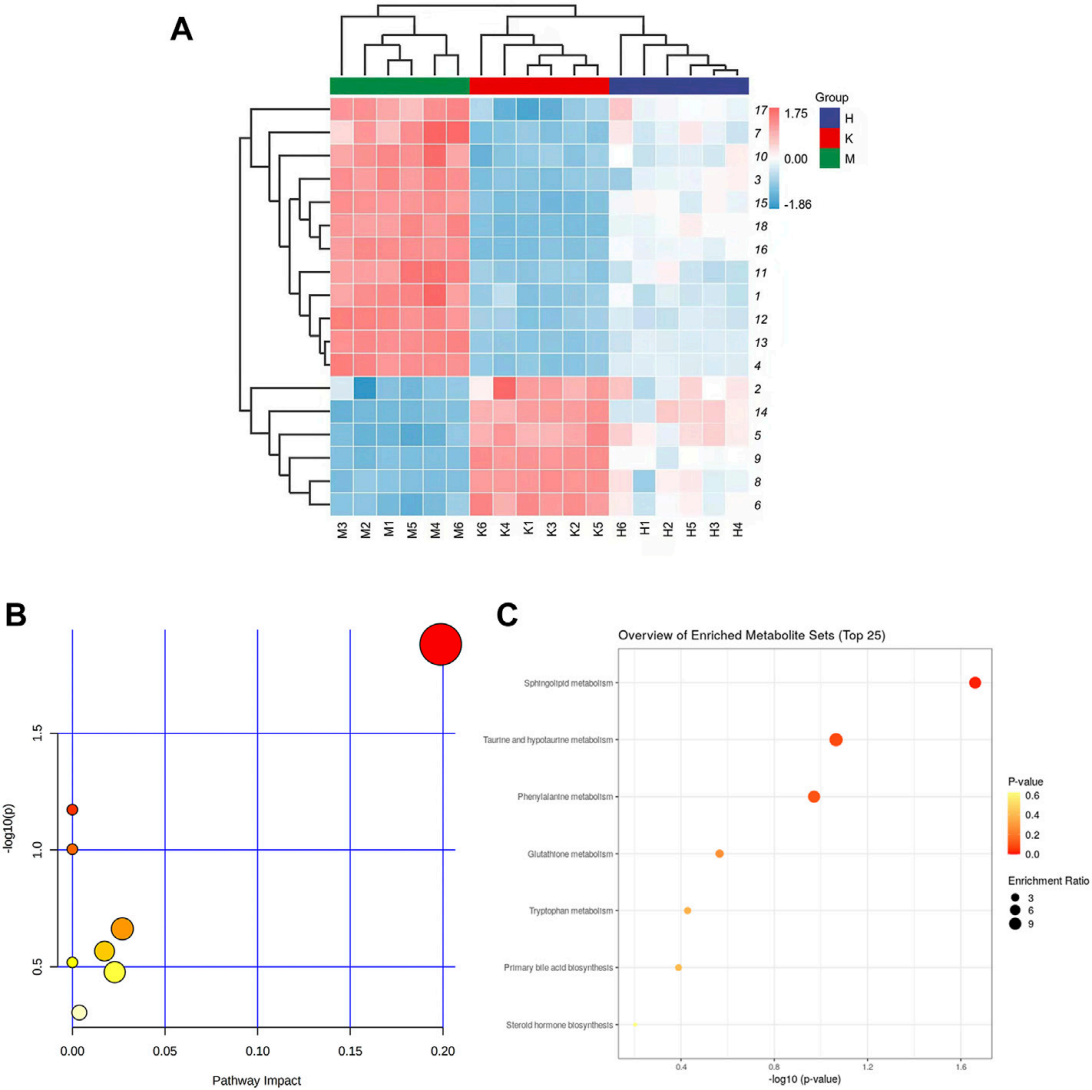
**(A)** Liver tissue metabolite cluster analysis diagram (The numbers on the right are the serial numbers of metabolites in the **Table 1**; **(B)** Metabolite enrichment analysis bubble diagram; **(C)** Metabolic pathway analysis diagram.

**FIGURE 4:**
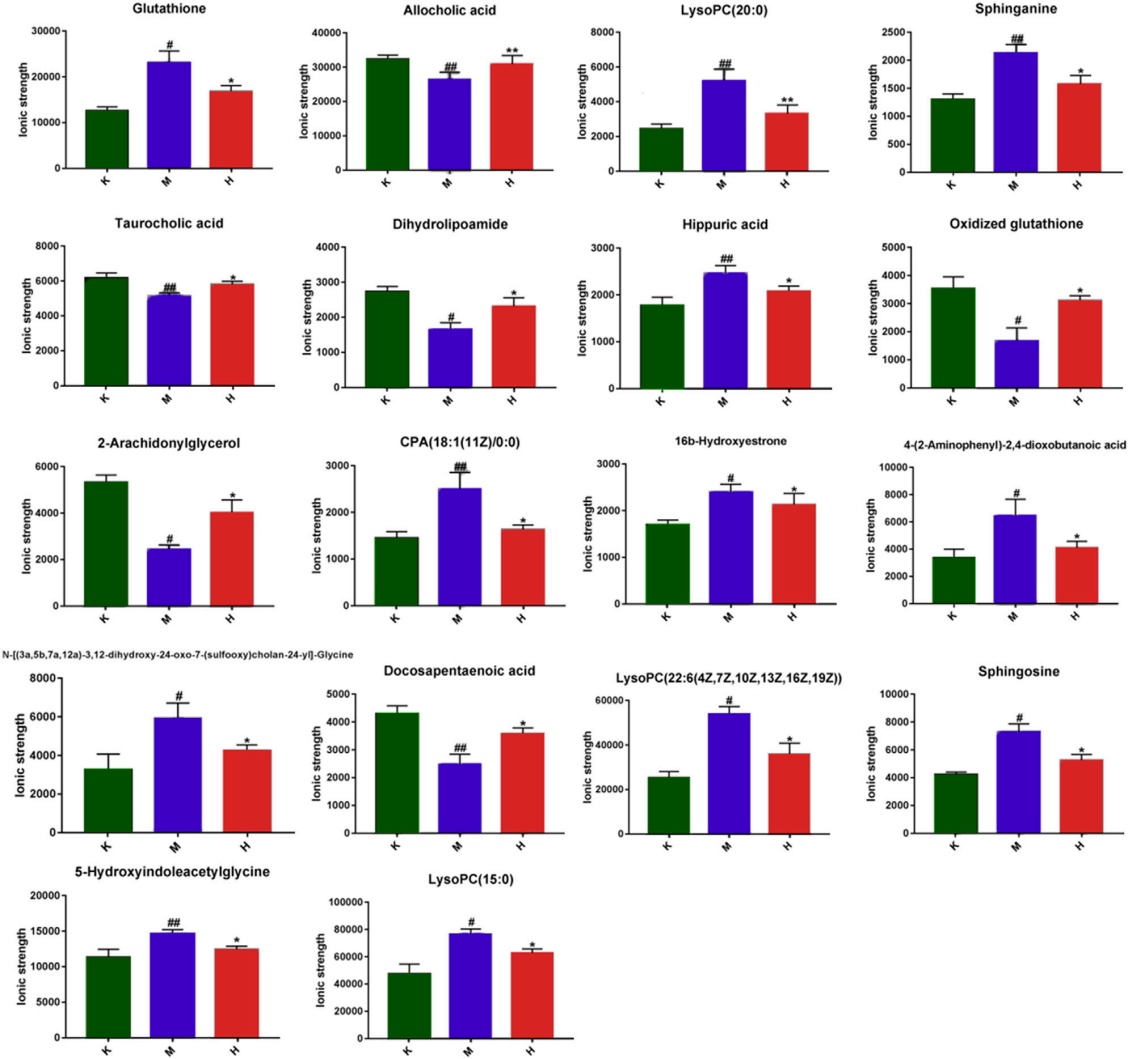
Impact values of potential biomarkers in the liver (Comparison with control group: ^**^ *p* < 0.01, ^*^ *p* < 0.05; Comparison with model group: ^##^ *p* < 0.01,^#^ *p* < 0.05).

The horizontal axis represents different experimental groups. The vertical axis represents biomarkers compared by groups. Each row represents different markers in different samples. Each column represents the expression level of all markers in each sample in the heat map of markers obtained by hierarchical cluster analysis, Figure 3A. The color blocks at various places show the relative expression of the position markers. The heat map intuitively depicts the content variations of 18 possible biomarkers between groups. Possible biomarkers in the normal and DLI model groups can be readily separated, as can potential biomarkers in the HQD and model groups. The content level of potential markers in the HQD group is closer to that of the normal control group than that of the model group, as indicated in Supplementary Table S1 and Figure 4.

The biomarkers selected from the HQD group were enriched in mice liver metabolomics using HMDB, KEGG, and other databases. The related metabolic pathways were identified using pathway analysis. Potential pathways should have a critical value greater than 0.10. The data indicate that it operates on eight different pathways, as shown in Figures 3B,C. As indicated in Supplementary Table S2, the major metabolic pathways with *p* < 0.05 and an effect value >0 include sphingolipid metabolism, taurine, and taurine metabolism, phenylalanine metabolism, glutathione metabolism, tryptophan metabolism, primary bile acid biosynthesis, and steroid hormone biosynthesis. Network pharmacological analysis.

**FIGURE 5:**
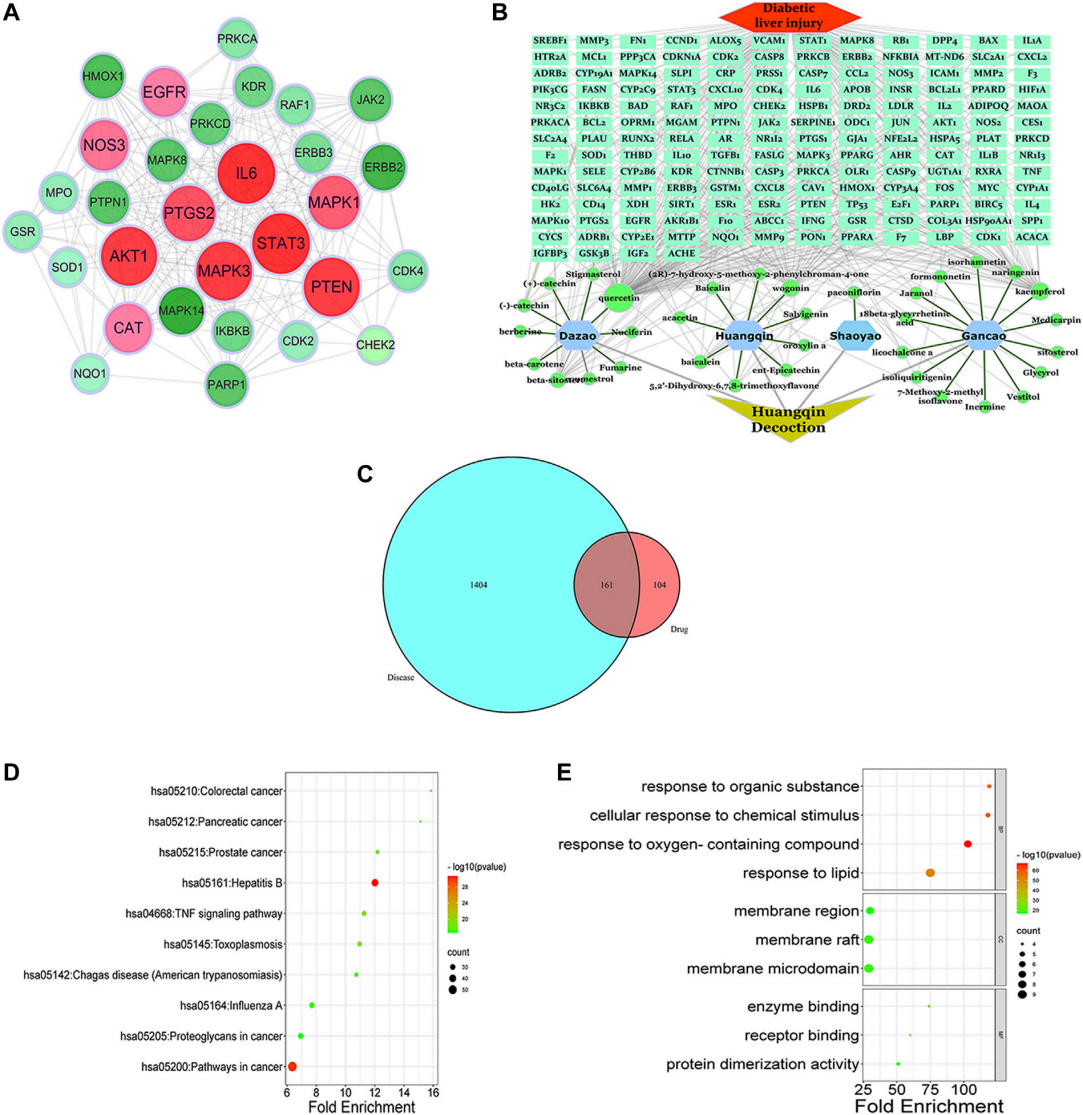
Network pharmacology analysis of HQD for treatment of DLI. **(A)** HQD and DLI intersection target Venn diagram; **(B)** HQD in the treatment of DLI component-target-disease interaction network; **(C)** PPI network diagram of HQD against DLI; **(D)** KEGG pathway enrichment analysis; **(E) **GO enrichment analysis of potential targets.

After entering the keyword “Scutellaria baicalensis, Paeonia alba, Jujube, and Licorice” into the TCMSP database, 34 effective components of Scutellaria baicalensis, eight effective components of Paeonia alba, 20 effective components of Jujube, and 90 effective components of Licorice were screened with OB ≥ 30% and DL ≥ 0.18. As shown in Supplementary Table S3, 141 components were obtained after merging and eliminating numerous elements. Obtain the SMILE standard structure of the active ingredients from the PubChem database (https://pubchem.ncbi.nlm.nih.gov/) and import it into the Swiss Target Prediction database (http://swisstargetprediction.ch/) for target prediction and deletion of duplicate targets. A total of 265 active ingredient-related targets have been obtained. Using the keyword “DLI,” 1404 potential disease targets were found in the GenBank, Genecards, and OMIM databases. Intersect the medicinal’ targets with the disease’s targets. We obtained 161 total targets of HQD and DLI after mapping (Supplementary Table S4) and drew a Venn diagram, Figures 5A. HQD’s “component-target-disease-pathway” network diagram is displayed in Cytoscape v3.8.2, Figures 5B.

Cytoscape 3.8.2 was used for visual analysis to construct a protein-protein interaction network to identify the essential genes of HQD against DLI, Figures 5–C. At the same time, the cytohubba plug-in was used to screen out the core targets, and when combined with the calculation method’s score, the top 10 genes (PTGS2, MAPK3, AKT1, MAPK1, PTEN, EGFR, STAT3, CAT, NOS3, IL6) are considered to be the central genes (see Supplementary Table S5). ClueGo was used to conduct GO annotation and KEGG pathway enrichment on the potential target genes of HQD against DLI Figures 5D,E. According to the GO enrichment analysis results, there is mainly response to an organic substance, cellular response to chemical stimulus, positive regulation of the metabolic process, positive regulation of Metabolic cellular process, and regulation of cell communication. According to KEGG enrichment analysis, pathways in cancer, Hepatitis B, Hepatitis C, PI3K-Akt signaling system, and AGE-RAGE signaling pathway in diabetic complications are all significantly affected.

### Integrative Analysis of the Network Pharmacology and Metabolomics

**FIGURE 6:**
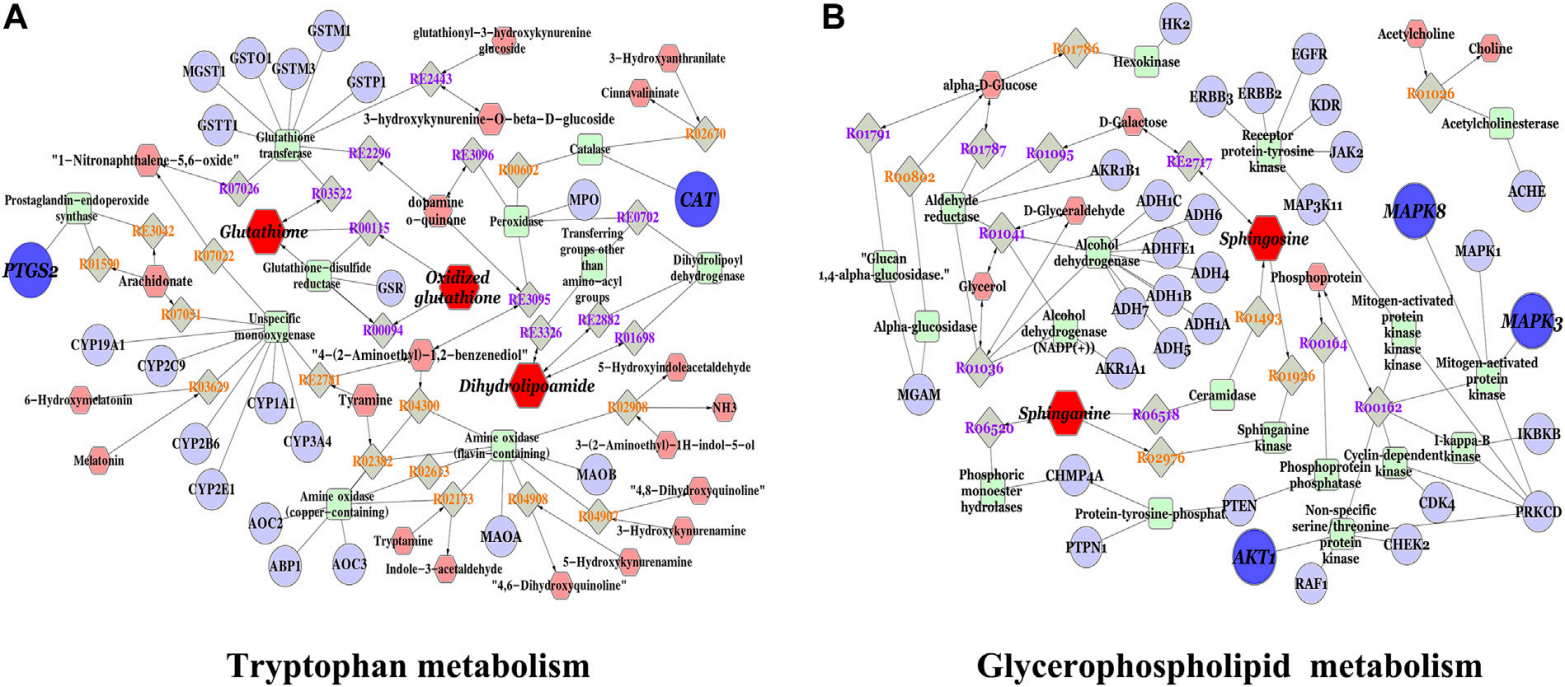
Compound-reaction-enzyme-gene network of key metabolites and targets; **(A)** Tryptophan metabolism; **(B)** Glyceropospholipid metabolism. Red hexagons, gray diamonds, green round rectangles, and purple circles represent active compounds, reactions, proteins, and genes, respectively.

We constructed an interaction network based on metabolomics and network pharmacology to completely understand the mechanism of HQD against DLI. The different metabolites were input into Cytoscape’s Metscape plug-in to generate a compound reaction enzyme gene network Figure 6. Sphingosine, glutathione, dihydrolipoamide, and oxidized glutathione are the major metabolites found in network pharmacology. Metscape analysis was used to connect potential targets with genes. We identified five important targets: CAT, PTGS2, MAPK3, AKT1, and MAPK8 (Supplementary Table S6). Glycerophospholipid metabolism; tryptophan metabolism is the effect pathways. They may have an important role in HQD’s therapeutic impact on DLI. Among these genes, PTGS2, MAPK3, and AKT1 are the key genes based on the degree values.

**FIGURE 7:**
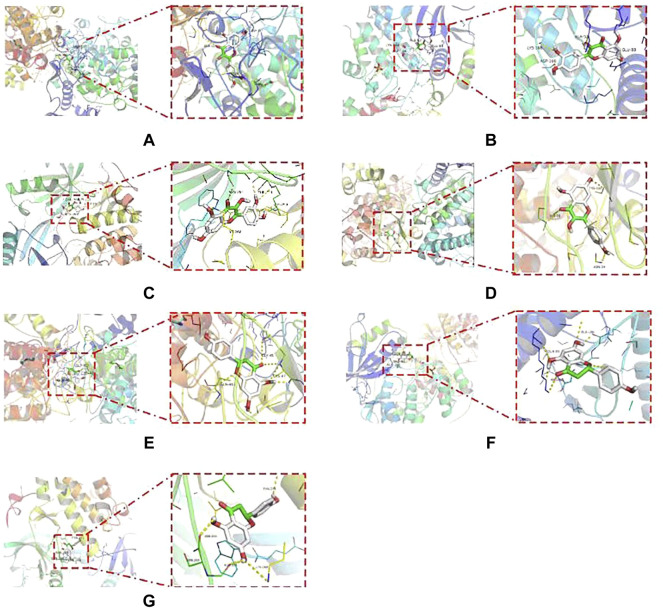
Molecular docking diagram of the first 7 positions of binding energy. **(A)** quercetin and PTGS2; **(B)** Quercetin and MAPK3; **(C)** Quercetin and AKT1; **(D)** Kaempferol and PTGS2; **(E)** Naringenin and PTGS2; **(F)** Naringenin and MAPK3; **(G)** Naringenin and AKT1.

After examining the RCSB protein database, molecular docking may investigate three main targets, further investigating the probability of interaction between the active components of HQD metformin and key targets. The binding energy is used to assess the strength of interactions between small molecules and proteins. If the binding energy is less than 0, it indicates that the ligand molecules may spontaneously bind to the receptor protein. If the binding energy is less than - 5.0 kcal⋅mol^−1^, it indicates that they have high binding activity, with the smaller the value indicating better binding activity. According to the molecular docking data, the binding energies of the key active components of HQD to AKT1, MAPK3, and PTGS2 were less than -5.0 kcal⋅mol^−1^ (Supplementary Table S7). The main active components (quercetin, kaempferol, wogonin, beta-sitosterol, naringenin) and target proteins (AKT1, MAPK3, PTGS2) in HQD have strong binding activity, according to the analysis of the results, and the docking structure of the first seven positions is shown in Figure 7.

## Discussion

### Confluence Analysis

Metabolomics technology analyzes low molecular weight endogenous metabolites in biological samples in detail, searches for new diagnostic or prognostic biomarkers of biological diseases, and explains the physiological changes associated with early diseases. On DLI, the metabolites and associated pathways of 18 different types of HQD were identified. On the other hand, Metabolomic investigations are limited to identify probable metabolites and linked pathways without further exploring their direct relationship. Network pharmacology is a systems biology-based methodology. It assesses the pharmacodynamic effects of medications at the molecular level to predict the interaction of natural products and proteins and identify the main mechanisms. Network pharmacology may help confirm the therapeutic management of metabolic networks and identify essential targets and biomarkers. Five important targets (CAT, PTGS2, MAPK3, AKT1, and MAPK8), five critical metabolites (sphinganine, sphingosine, Glutahione, Oxidized gutahione, Dihydrolipoamide), and two linked metabolic pathways (glycerol phospholipid metabolism and tryptophan metabolism) were identified by integrating metabolomics with network pharmacology.

Related pharmacodynamic studies have shown the potential mechanism of HQD in the treatment of DLI. It was shown that HQD might considerably lower blood AST and ALT levels and MDA activity in the liver of model group, increase SOD activity in the liver and alleviate the degree of pathological damage to liver tissue. Endogenous compounds are bound to change in the liver, which is an important organ of human metabolism, and variations in its composition might reflect the entire metabolic profile of the body. The current study used liver metabolomics to investigate the changes in metabolites in the liver of mice and the protective impact of HQD on mice with DLI metabolic disorders from a metabolomics approach.

### Lipid Metabolism

Glycerophospholipid metabolism is a form of lipid metabolism, and glycerophospholipids are the most abundant type of phospholipids in the body, being extensively dispersed throughout the biological membranes of numerous organs. The formation and degradation of glycerophospholipid molecules are related to lipid transport throughout the body (Kagan et al., 2016). Phosphatidylcholine, phosphatidylethanolamine, sphingolipids, and lysophosphatidylcholine are the most important lipid metabolites in the metabolism of glycerophospholipids. These lipids are essential components of serum lipoproteins and cell membranes, and they play a role in signal transmission. Through the hydrolysis of phospholipases, the metabolism of glycerophospholipids connects the important signal pathways of bodily fluid metabolism, supports the proper operation of material and energy metabolism, and maintains the balance and steady-state of metabolism. Phospholipase synthesis is another critical connection that influences glycerophospholipid metabolites. If its content is decreased, many phospholipid metabolites will accumulate, resulting in the formation of associated metabolic disorders (Shen et al., 2020). According to studies, DLI may cause glycerophospholipid molecules to be unable to be transported properly throughout the liver, resulting in deposits. Excessive glycerophospholipid deposits, on the other hand, might harm the liver. The glycerophospholipid metabolism in the liver of DLI mice was disrupted in this study, and HQD had a considerable restorative impact on the glycerophospholipid metabolism in DLI mice. In DLI mice, lysophosphatidylcholine and sphingolipid levels were disordered, LysoPC(15:0), LysoPC(20:0), LysoPC(22:6 (4Z,7Z,10Z,13Z,16Z, 19Z)), sphinganine, and sphingosine levels rose, and HQD had a positive impact on this. When combined with network analysis, it is hypothesized that HQD may regulate the levels of LysoPC (15:0), LysoPC(20:0), LysoPC (22:6 (4Z,7Z,10Z,13,16Z, 19Z)) and the levels of sphingolipids sphinganine and sphingosine, thus also regulating the glycerophospholipid metabolism network and achieving disease prevention and treatment. The molecular docking studies demonstrate that the core active components of HQD have a strong binding capacity with AKT and MAPK3, suggesting that HQD may impact AKT1 and MAPK3 Activity, which in turn affects glycerophospholipid metabolism.

### Amino Acid Metabolism

As an important component of the human body, amino acids play a critical role in the metabolic pathways of numerous substances. As the basic substance of energy metabolism, the amino acid is a component of protein and an active substance with regulatory effects in a wide range of life activities. The liver is the main organ of material metabolism. It is essential for amino acid metabolism, protein synthesis, and breakdown. Tryptophan is an important amino acid for the human body. It can synthesize proteins and metabolize them into a range of bioactive molecules (Myint et al., 2013). It is a precursor of the neurotransmitter serotonin, which is employed for protein synthesis or converted into a range of bioactive compounds through the serotonin or kynurenine pathways (KP). The variety of physiological processes controlled by tryptophan reflects the complicated pattern of diseases associated with homeostasis alterations (Comai et al., 2020). Studies have shown that tryptophan metabolism disorder is found in diabetes and psychiatric disorders (Gulaj et al., 2010). During metabolism, tryptophan may be transformed into acetyl coenzyme A and subsequently into acetyl coenzyme A, entering the tricarboxylic acid cycle. It is created during the tricarboxylic acid cycle metabolism α-ketoglutarate, pyruvate, and oxaloacetic acid may be converted into corresponding amino acids by interacting with appropriate ammonia, such as glutamate, aspartic acid, alanine, and so on. Under the control of glutamylcysteine synthase and glutathione synthase, glutamic acid, cysteine, and glycine are condensed into glutathione through peptide bonds (Mutlib et al., 2000; Salyha, 2013; Gong et al., 2020; Xiang et al., 2021). This investigation revealed that, compared to the model group, the HQD treatment group could considerably callback the glutathione and oxidized glutathione concentrations. When combined with network analysis, it is hypothesized that HQD may indirectly restore tryptophan metabolism by controlling glutathione and oxidized glutathione levels, so working as disease prevention and therapy. Further molecular docking research showed that HQD might indirectly alter neurotransmitter production and tryptophan metabolism by changing PTGS2 activity.

## Conclusion

The combination of network pharmacology and metabolomics analysis in this study can help explain the possible mechanism of HQD in treating DLI and reveal the biological process of HQD in treating DLI through regulating metabolic pathways. A detailed analysis revealed five main targets (CAT, PTGS2, MAPK3, AKT1, and MAPK8), as well as related metabolites sphinganine, sphingosine, Glutahione, Oxidized gutahione, Dihydrolipoamide and pathways Glycerol phospholipid metabolism and Tryptophan metabolism. Molecular docking was used to confirm these targets. This research provides evidence and theoretical support for future investigation of the mechanism and establishes a framework for clinical application. It is expected to contribute to the development of HQD as a potential complementary medicine for treating DLI. More molecular biological studies are required to confirm the detailed mechanism.

## Data Availability

The original contributions presented in the study are included in the article/Supplementary Material, further inquiries can be directed to the corresponding authors.

## Supplementary Material

Supplementary Figure S1 Determination of baicalin and paeoniflorin by HPLC.
