# Supplementary material for: Integrating Network Pharmacology and Metabolomics to Elucidate the Mechanism of Action of Huang Qin Decoction for Treament of Diabetic Liver Injury
Source: Front Pharmacol. 2022 May 25;13:899043. doi: 10.3389/fphar.2022.899043 (PMC9176298; doi:10.3389/fphar.2022.899043)
Supplement: Supplementary file 1 [file Table1.docx]

**Table 1 Potential biomarkers in DLD mice liver**

| No. | ESI  mode | Metabolites | Chemical  formula | Rt-m/z | HMDB | KEGG | Control/Model | Model/H |
| --- | --- | --- | --- | --- | --- | --- | --- | --- |
| 1 | neg | Glutathione | C10H17N3O6S | 0.81_307.0833n | HMDB0000125 | C00051 | ^↑*^ | ^↓#^ |
| 2 | neg | Allocholic acid | C24H40O5 | 2.87_453.2948m/z | HMDB0000505 | C17737 | ↓^**^ | ↑^##^ |
| 3 | pos | LysoPC(20:0) | C28H58NO7P | 7.49_552.4048m/z | HMDB0010390 | C04230 | ^↑**^ | ^↓#^ |
| 4 | pos | Sphinganine | C18H39NO2 | 4.04_302.3027m/z | HMDB0000269 | C00836 | ↑^*^ | ↓^#^ |
| 5 | pos | Taurocholic acid | C26H45NO7S | 2.49_1031.5534m/z | HMDB0000036 | C05122 | ↓^**^ | ↑^#^ |
| 6 | pos | Dihydrolipoamide | C8H17NOS2 | 1.62_190.0919m/z | HMDB0000985 | C00579 | ↓^*^ | ↑^#^ |
| 7 | pos | Hippuric acid | C9H9NO3 | 1.24_162.0609m/z | HMDB0000714 | C01586 | ↑^**^ | ↓^#^ |
| 8 | pos | Oxidized glutathione | C20H32N6O12S2 | 1.15_613.1653m/z | HMDB0003337 | C00127 | ^↓*^ | ^↑#^ |
| 9 | pos | 2-Arachidonylglycerol | C23H38O4 | 4.32_361.2692m/z | HMDB0004666 | C13856 | ^↓*^ | ^↑#^ |
| 10 | pos | CPA(18:1(11Z)/0:0) | C21H39O6P | 4.35_401.2427m/z | HMDB0007005 | - | ↑^**^ | ↓^#^ |
| 11 | pos | 16b-Hydroxyestrone | C18H22O3 | 5.64_573.3062m/z | HMDB0000313 | C05300 | ^↑*^ | ^↓#^ |
| 12 | pos | 4-(2-Aminophenyl)-2,4-dioxobutanoic acid | C10H9NO4 | 5.42_190.0525m/z | HMDB0000978 | C01252 | ^↓*^ | ^↑#^ |
| 13 | pos | N-[(3a,5b,7a,12a)-3,12-dihydroxy-24-oxo-7-(sulfooxy)cholan-24-yl]-Glycine | C26H43NO9S | 4.45_546.2878m/z | HMDB0002640 | - | ^↑*^ | ^↓#^ |
| 14 | pos | Docosapentaenoic acid | C22H34O2 | 5.02_313.2703m/z | HMDB0006528 | C16513 | ↓^**^ | ↑^#^ |
| 15 | pos | LysoPC(22:6(4Z,7Z,10Z,13Z,16Z,19Z)) | C30H50NO7P | 4.30_567.3378n | HMDB0010404 | C04230 | ↑^*^ | ↓^#^ |
| 16 | pos | Sphingosine | C18H37NO2 | 3.82_299.2808n | HMDB0000252 | C05832 | ^↑**^ | ^↓#^ |
| 17 | pos | 5-Hydroxyindoleacetylglycine | C12H12N2O4 | 2.08_248.0887n | HMDB0004185 | C05832 | ↓^**^ | ↑^#^ |
| 18 | pos | LysoPC(15:0) | C23H48NO7P | 6.14_481.3131n | HMDB0010381 | C04230 | ↑^*^ | ↓^#^ |
