# Supplementary material for: Integrating Network Pharmacology and Metabolomics to Elucidate the Mechanism of Action of Huang Qin Decoction for Treament of Diabetic Liver Injury
Source: Front Pharmacol. 2022 May 25;13:899043. doi: 10.3389/fphar.2022.899043 (PMC9176298; doi:10.3389/fphar.2022.899043)
Supplement: Supplementary file 2 [file Table7.docx]

**Table 7 Molecular docking results**

| Compound | Docking score(kcal/mol) | | |
| --- | --- | --- | --- |
|  | AKT1 | MAPK3 | PTGS2 |
| quercetin | -10.5 | -8.1 | -8.4 |
| kaempferol | -7.3 | -8.0 | -8.9 |
| wogonin | -9.7 | -7.7 | -8.5 |
| beta-sitosterol | -6 | -5.9 | -5.9 |
| naringenin | -9.8 | -8.8 | -9.5 |
| Metformin | -5.9 | -4.9 | -5.6 |
