# Supplementary figures and images for: Integrating Network Pharmacology and Metabolomics to Elucidate the Mechanism of Action of Huang Qin Decoction for Treament of Diabetic Liver Injury
Source: Front Pharmacol. 2022 May 25;13:899043. doi: 10.3389/fphar.2022.899043 (PMC9176298; doi:10.3389/fphar.2022.899043)

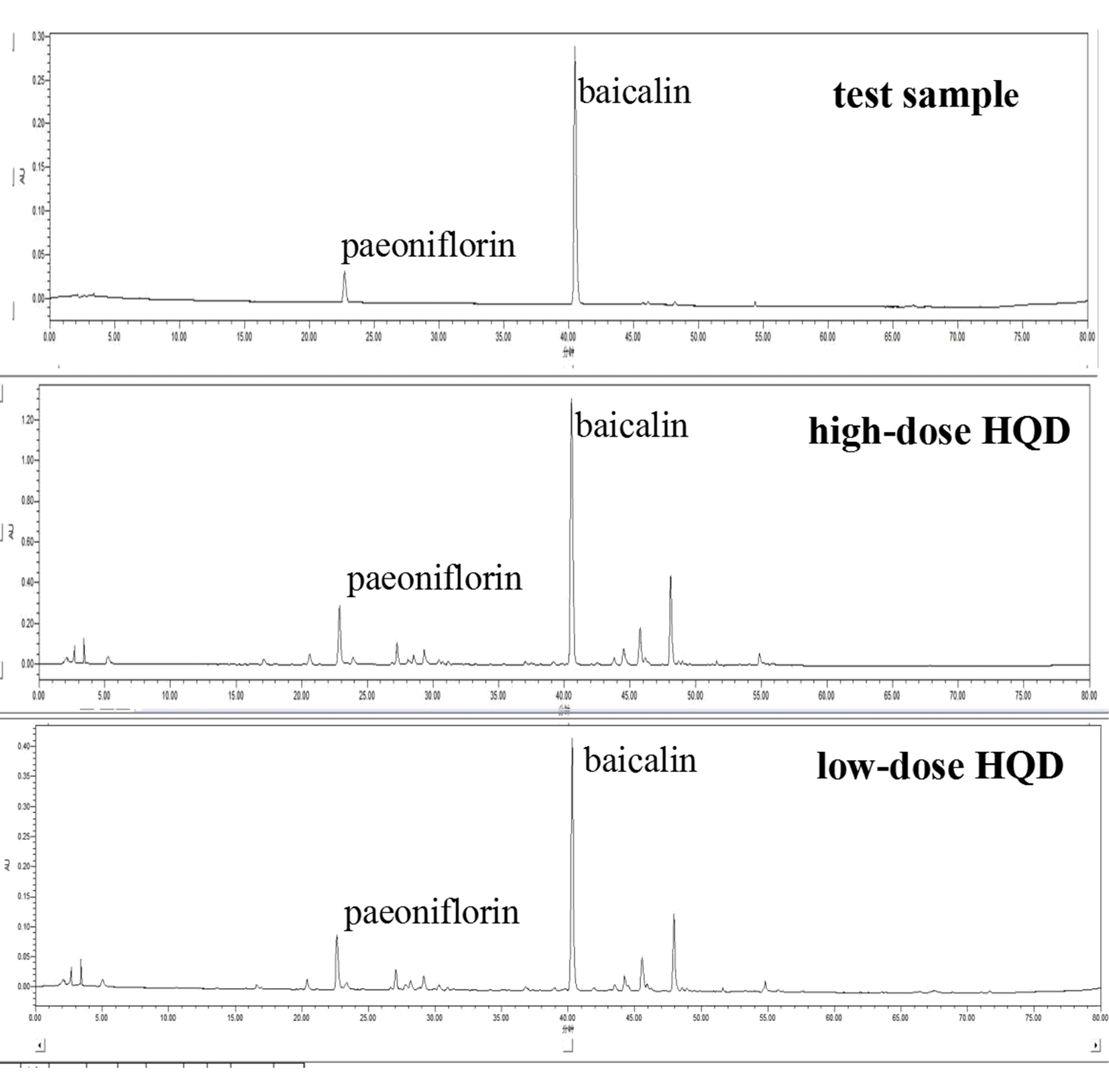

Supplement: Supplementary file 3 [file Image1.tif]
