# Supplementary material for: Integrating Network Pharmacology and Metabolomics to Elucidate the Mechanism of Action of Huang Qin Decoction for Treament of Diabetic Liver Injury
Source: Front Pharmacol. 2022 May 25;13:899043. doi: 10.3389/fphar.2022.899043 (PMC9176298; doi:10.3389/fphar.2022.899043)
Supplement: Supplementary file 4 [file Table2.docx]

**Table 2** **Metabolic pathways regulated by HQD**

| Pathway name | Total | Expected | Hits | Raw p | -LOG | Holm adjust | FDR | Impact |
| --- | --- | --- | --- | --- | --- | --- | --- | --- |
| Sphingolipid metabolism | 21 | 0.18127 | 2 | 0.013173 | 1.8803 | 1 | 1 | 0.19878 |
| Taurine and hypotaurine metabolism | 8 | 0.069057 | 1 | 0.067158 | 1.1729 | 1 | 1 | 0 |
| Phenylalanine metabolism | 12 | 0.10359 | 1 | 0.099152 | 1.0037 | 1 | 1 | 0 |
| Glutathione metabolism | 28 | 0.2417 | 1 | 0.21727 | 0.66301 | 1 | 1 | 0.02698 |
| Glycerophospholipid metabolism | 36 | 0.31076 | 1 | 0.2708 | 0.56735 | 1 | 1 | 0.01736 |
| Tryptophan metabolism | 41 | 0.35392 | 1 | 0.30252 | 0.51925 | 1 | 1 | 0 |
| Primary bile acid biosynthesis | 46 | 0.39708 | 1 | 0.33296 | 0.4776 | 1 | 1 | 0.02285 |
| Steroid hormone biosynthesis | 77 | 0.66467 | 1 | 0.49595 | 0.30456 | 1 | 1 | 0.00377 |
