# Supplementary material for: Integrating Network Pharmacology and Metabolomics to Elucidate the Mechanism of Action of Huang Qin Decoction for Treament of Diabetic Liver Injury
Source: Front Pharmacol. 2022 May 25;13:899043. doi: 10.3389/fphar.2022.899043 (PMC9176298; doi:10.3389/fphar.2022.899043)
Supplement: Supplementary file 5 [file Table5.docx]

**Table 5 Key genes for CytoHubba screening**

| Gene symbol | Rank methods in CytoHubba | | | | | | | | | |
| --- | --- | --- | --- | --- | --- | --- | --- | --- | --- | --- |
|  | MCC | MNC | Degree | EPC | BottleNeck | EcCentricity | Closeness | Radiality | Betweenness | Stress |
| AKT1 | 9.22E+13 | 100 | 200 | 26.089 | 2 | 0.5 | 130 | 2.65 | 526.37295 | 46488 |
| CAT | 9.22E+13 | 84 | 168 | 22.955 | 1 | 0.5 | 122 | 2.55 | 363.04732 | 38904 |
| EGFR | 9.22E+13 | 61 | 122 | 20.721 | 1 | 0.5 | 110.5 | 2.40625 | 81.61511 | 9752 |
| IL6 | 9.22E+13 | 39 | 78 | 15.73 | 1 | 0.33333 | 99 | 2.25 | 19.80374 | 4040 |
| MAPK1 | 9.22E+13 | 36 | 72 | 12.257 | 1 | 0.33333 | 97.83333 | 2.24375 | 65.86494 | 6456 |
| MAPK3 | 9.22E+13 | 35 | 70 | 12.125 | 1 | 0.33333 | 97.33333 | 2.2375 | 65.82746 | 8440 |
| NOS3 | 9.22E+13 | 27 | 54 | 11.104 | 1 | 0.33333 | 91.33333 | 2.1125 | 4.22634 | 528 |
| PTEN | 5.67E+08 | 18 | 36 | 7.267 | 1 | 0.33333 | 86 | 2.025 | 4.91055 | 432 |
| PTGS2 | 7299390 | 16 | 32 | 6.973 | 1 | 0.33333 | 87.33333 | 2.1 | 20.67238 | 1568 |
| STAT3 | 10128 | 9 | 18 | 4.061 | 1 | 0.33333 | 80.5 | 1.93125 | 0.53376 | 56 |
