# Supplementary material for: Integrating Network Pharmacology and Metabolomics to Elucidate the Mechanism of Action of Huang Qin Decoction for Treament of Diabetic Liver Injury
Source: Front Pharmacol. 2022 May 25;13:899043. doi: 10.3389/fphar.2022.899043 (PMC9176298; doi:10.3389/fphar.2022.899043)
Supplement: Supplementary file 6 [file Table3.docx]

**Table 3 HQD active ingredients**

| **MOL ID** | **ingredients** | **OB（%）** | **DL** | **Source** |
| --- | --- | --- | --- | --- |
| MOL000422 | kaempferol | 41.88224954 | 0.24066 | Licorice、 Radix Paeoniae Alba |
| MOL000359 | sitosterol | 36.91390583 | 0.7512 | Licorice、 Radix Scutellariae 、Radix Paeoniae Alba |
| MOL000098 | quercetin | 46.43334812 | 0.27525 | Jujube、licorice |
| MOL000211 | Mairin | 55.37707338 | 0.7761 | Jujube、licorice、 Radix Paeoniae Alba |
| MOL000358 | beta-sitosterol | 36.91390583 | 0.75123 | Jujube、 Radix Scutellariae 、Radix Paeoniae Alba |
| MOL000449 | Stigmasterol | 43.82985158 | 0.75665 | Jujube、 Radix Scutellariae |
| MOL000492 | (+)-catechin | 54.82643405 | 0.24164 | Jujube、Radix Paeoniae Alba |
| MOL000096 | (-)-catechin | 49.6763868 | 0.24162 | Jujube |
| MOL000627 | Stepholidine | 33.10625074 | 0.54083 | Jujube |
| MOL000787 | Fumarine | 59.26250458 | 0.82694 | Jujube |
| MOL001454 | berberine | 36.86124504 | 0.77665 | Jujube |
| MOL001522 | (S)-Coclaurine | 42.35064217 | 0.23518 | Jujube |
| MOL002773 | beta-carotene | 37.18433337 | 0.58358 | Jujube |
| MOL004350 | Ruvoside_qt | 36.12101953 | 0.75671 | Jujube |
| MOL007213 | Nuciferin | 34.43102883 | 0.40475 | Jujube |
| MOL012921 | stepharine | 31.54786691 | 0.33376 | Jujube |
| MOL012946 | zizyphus saponin I_qt | 32.69113507 | 0.61923 | Jujube |
| MOL012976 | coumestrol | 32.48702929 | 0.33733 | Jujube |
| MOL012981 | Daechuine S7 | 44.81774487 | 0.82806 | Jujube |
| MOL012986 | Jujubasaponin V_qt | 36.98963109 | 0.63448 | Jujube |
| MOL012992 | Mauritine D | 89.12509381 | 0.45286 | Jujube |
| MOL000263 | oleanolic acid | 29.02 | 0.76 | Jujube |
| MOL000354 | isorhamnetin | 49.60437705 | 0.306 | Licorice |
| MOL003656 | Lupiwighteone | 51.63569181 | 0.36739 | Licorice |
| MOL004808 | glyasperin B | 65.22438608 | 0.43851 | Licorice |
| MOL004810 | glyasperin F | 75.83680013 | 0.53514 | Licorice |
| MOL004811 | Glyasperin C | 45.56380662 | 0.39947 | Licorice |
| MOL004820 | kanzonols W | 50.48007599 | 0.51704 | Licorice |
| MOL004824 | (2S)-6-(2,4-dihydroxyphenyl)-2-(2-hydroxypropan-2-yl)-4-methoxy-2,3-dihydrofuro[3,2-g]chromen-7-one | 60.25040908 | 0.63433 | Licorice |
| MOL004827 | Semilicoisoflavone B | 48.77755194 | 0.54732 | Licorice |
| MOL004828 | Glepidotin A | 44.72187465 | 0.34685 | Licorice |
| MOL004829 | Glepidotin B | 64.46292386 | 0.34485 | Licorice |
| MOL004849 | 3-(2,4-dihydroxyphenyl)-8-(1,1-dimethylprop-2-enyl)-7-hydroxy-5-methoxy-coumarin | 59.62247498 | 0.42894 | Licorice |
| MOL004855 | Licoricone | 63.57845938 | 0.4712 | Licorice |
| MOL004856 | Gancaonin A | 51.07519107 | 0.40378 | Licorice |
| MOL004857 | Gancaonin B | 48.79440201 | 0.44924 | Licorice |
| MOL004864 | 5,7-dihydroxy-3-(4-methoxyphenyl)-8-(3-methylbut-2-enyl)chromone | 30.48877673 | 0.41002 | Licorice |
| MOL004879 | Glycyrin | 52.60657166 | 0.47466 | Licorice |
| MOL004883 | Licoisoflavone | 41.61021885 | 0.41646 | Licorice |
| MOL004884 | Licoisoflavone B | 38.92870888 | 0.54714 | Licorice |
| MOL004885 | licoisoflavanone | 52.46624706 | 0.54488 | Licorice |
| MOL004904 | licopyranocoumarin | 80.36001331 | 0.6535 | Licorice |
| MOL004959 | 1-Methoxyphaseollidin | 36.56537233 | 0.32291 | Licorice |
| MOL004966 | 3'-Hydroxy-4'-O-Methylglabridin | 43.71495141 | 0.57406 | Licorice |
| MOL004974 | 3'-Methoxyglabridin | 46.16150929 | 0.57393 | Licorice |
| MOL005000 | Gancaonin G | 60.43520506 | 0.39404 | Licorice |
| MOL005001 | Gancaonin H | 50.10372327 | 0.78416 | Licorice |
| MOL005007 | Glyasperins M | 72.67080984 | 0.59274 | Licorice |
| MOL005008 | Glycyrrhiza flavonol A | 41.27527733 | 0.59512 | Licorice |
| MOL000497 | licochalcone a | 40.78965199 | 0.28517 | Licorice |
| MOL004328 | naringenin | 59.29389773 | 0.21128 | Licorice |
| MOL004903 | liquiritin | 65.69011165 | 0.73893 | Licorice |
| MOL000392 | formononetin | 69.67388061 | 0.21202 | Licorice |
| MOL000500 | Vestitol | 74.65518912 | 0.20935 | Licorice |
| MOL001792 | DFV | 32.76272375 | 0.18316 | Licorice |
| MOL002565 | Medicarpin | 49.21981761 | 0.3351 | Licorice |
| MOL003896 | 7-Methoxy-2-methyl isoflavone | 42.56474148 | 0.19946 | Licorice |
| MOL004835 | Glypallichalcone | 61.59706227 | 0.18993 | Licorice |
| MOL004941 | (2R)-7-hydroxy-2-(4-hydroxyphenyl)chroman-4-one | 71.12298901 | 0.18303 | Licorice |
| MOL004957 | HMO | 38.3654238 | 0.21067 | Licorice |
| MOL004978 | 2-[(3R)-8,8-dimethyl-3,4-dihydro-2H-pyrano[6,5-f]chromen-3-yl]-5-methoxyphenol | 36.21429208 | 0.52122 | Licorice |
| MOL000239 | Jaranol | 50.82881677 | 0.29148 | Licorice |
| MOL001484 | Inermine | 75.18306038 | 0.53754 | Licorice |
| MOL004806 | euchrenone | 30.28726099 | 0.57386 | Licorice |
| MOL004815 | (E)-1-(2,4-dihydroxyphenyl)-3-(2,2-dimethylchromen-6-yl)prop-2-en-1-one | 39.61685537 | 0.35077 | Licorice |
| MOL004833 | Phaseolinisoflavan | 32.00810772 | 0.44538 | Licorice |
| MOL004866 | 2-(3,4-dihydroxyphenyl)-5,7-dihydroxy-6-(3-methylbut-2-enyl)chromone | 44.15196126 | 0.41482 | Licorice |
| MOL004891 | shinpterocarpin | 80.29527688 | 0.72746 | Licorice |
| MOL004908 | Glabridin | 53.24514328 | 0.46967 | Licorice |
| MOL004910 | Glabranin | 52.89565508 | 0.31208 | Licorice |
| MOL004911 | Glabrene | 46.26685721 | 0.43902 | Licorice |
| MOL004912 | Glabrone | 52.51217419 | 0.49645 | Licorice |
| MOL004915 | Eurycarpin A | 43.27728425 | 0.37429 | Licorice |
| MOL004945 | (2S)-7-hydroxy-2-(4-hydroxyphenyl)-8-(3-methylbut-2-enyl)chroman-4-one | 36.56537233 | 0.32291 | Licorice |
| MOL004961 | Quercetin der. | 46.4493884 | 0.3343 | Licorice |
| MOL004980 | Inflacoumarin A | 39.70909598 | 0.32613 | Licorice |
| MOL004989 | 6-prenylated eriodictyol | 39.22383018 | 0.41259 | Licorice |
| MOL004991 | 7-Acetoxy-2-methylisoflavone | 38.92333105 | 0.26217 | Licorice |
| MOL004993 | 8-prenylated eriodictyol | 53.79476318 | 0.40383 | Licorice |
| MOL005003 | Licoagrocarpin | 58.81390287 | 0.58498 | Licorice |
| MOL005012 | Licoagroisoflavone | 57.28224098 | 0.48679 | Licorice |
| MOL005016 | Odoratin | 49.94821817 | 0.30487 | Licorice |
| MOL005020 | dehydroglyasperins C | 53.82326014 | 0.37006 | Licorice |
| MOL000417 | Calycosin | 47.75182783 | 0.24278 | Licorice |
| MOL004838 | 8-(6-hydroxy-2-benzofuranyl)-2,2-dimethyl-5-chromenol | 58.43728091 | 0.38106 | Licorice |
| MOL004863 | 3-(3,4-dihydroxyphenyl)-5,7-dihydroxy-8-(3-methylbut-2-enyl)chromone | 66.37125046 | 0.41392 | Licorice |
| MOL002311 | Glycyrol | 90.77578223 | 0.66819 | Licorice |
| MOL004805 | (2S)-2-[4-hydroxy-3-(3-methylbut-2-enyl)phenyl]-8,8-dimethyl-2,3-dihydropyrano[2,3-f]chromen-4-one | 31.78703353 | 0.72403 | Licorice |
| MOL004814 | Isotrifoliol | 31.94478724 | 0.42422 | Licorice |
| MOL004841 | Licochalcone B | 76.75735485 | 0.1935 | Licorice |
| MOL004848 | licochalcone G | 49.25496332 | 0.32325 | Licorice |
| MOL004898 | (E)-3-[3,4-dihydroxy-5-(3-methylbut-2-enyl)phenyl]-1-(2,4-dihydroxyphenyl)prop-2-en-1-one | 46.26792256 | 0.3062 | Licorice |
| MOL004907 | Glyzaglabrin | 61.06888631 | 0.35347 | Licorice |
| MOL004924 | (-)-Medicocarpin | 40.99397199 | 0.95059 | Licorice |
| MOL004935 | Sigmoidin-B | 34.88108616 | 0.41455 | Licorice |
| MOL004948 | Isoglycyrol | 44.69922568 | 0.83845 | Licorice |
| MOL004949 | Isolicoflavonol | 45.16999058 | 0.41859 | Licorice |
| MOL004988 | Kanzonol F | 32.46833364 | 0.89364 | Licorice |
| MOL004990 | 7,2',4'-trihydroxy－5-methoxy-3－arylcoumarin | 83.71436744 | 0.27136 | Licorice |
| MOL005017 | Phaseol | 78.76621925 | 0.57867 | Licorice |
| MOL005018 | Xambioona | 54.84916242 | 0.87419 | Licorice |
| MOL004913 | 1,3-dihydroxy-9-methoxy-6-benzofurano[3,2-c]chromenone | 48.14154235 | 0.42831 | Licorice |
| MOL004914 | 1,3-dihydroxy-8,9-dimethoxy-6-benzofurano[3,2-c]chromenone | 62.90135486 | 0.52759 | Licorice |
| MOL004985 | icos-5-enoic acid | 30.70294255 | 0.19725 | Licorice |
| MOL004996 | gadelaidic acid | 30.70294255 | 0.19725 | Licorice |
| MOL004882 | Licocoumarone | 33.21085068 | 0.3568 | Licorice |
| MOL001789 | isoliquiritigenin | 85.32 | 0.15 | Licorice |
| MOL004804 | 18beta-glycyrrhetinic acid | 22.05 | 0.74 | Licorice |
| MOL000073 | ent-Epicatechin | 48.95984114 | 0.24162 | Radix Scutellariae |
| MOL000173 | wogonin | 30.68456706 | 0.22942 | Radix Scutellariae |
| MOL000228 | (2R)-7-hydroxy-5-methoxy-2-phenylchroman-4-one | 55.23317389 | 0.20163 | Radix Scutellariae |
| MOL000525 | Norwogonin | 39.40397184 | 0.20723 | Radix Scutellariae |
| MOL000552 | 5,2'-Dihydroxy-6,7,8-trimethoxyflavone | 31.71246493 | 0.35462 | Radix Scutellariae |
| MOL001458 | coptisine | 30.671852 | 0.85647 | Radix Scutellariae |
| MOL001490 | bis[(2S)-2-ethylhexyl] benzene-1,2-dicarboxylate | 43.59332547 | 0.34531 | Radix Scutellariae |
| MOL001689 | acacetin | 34.97357273 | 0.24082 | Radix Scutellariae |
| MOL002714 | baicalein | 33.51891869 | 0.20888 | Radix Scutellariae |
| MOL002879 | Diop | 43.59332547 | 0.39247 | Radix Scutellariae |
| MOL002897 | epiberberine | 43.09233228 | 0.7761 | Radix Scutellariae |
| MOL002909 | 5,7,2,5-tetrahydroxy-8,6-dimethoxyflavone | 33.81582599 | 0.44739 | Radix Scutellariae |
| MOL002910 | Carthamidin | 41.15096273 | 0.24189 | Radix Scutellariae |
| MOL002913 | Dihydrobaicalin_qt | 40.03778103 | 0.20722 | Radix Scutellariae |
| MOL002914 | Eriodyctiol (flavanone) | 41.35042713 | 0.2436 | Radix Scutellariae |
| MOL002915 | Salvigenin | 49.06592606 | 0.33279 | Radix Scutellariae |
| MOL002917 | 5,2',6'-Trihydroxy-7,8-dimethoxyflavone | 45.04742802 | 0.33057 | Radix Scutellariae |
| MOL002925 | 5,7,2',6'-Tetrahydroxyflavone | 37.01348688 | 0.24382 | Radix Scutellariae |
| MOL002927 | Skullcapflavone II | 69.51043398 | 0.4379 | Radix Scutellariae |
| MOL002928 | oroxylin a | 41.367569 | 0.23233 | Radix Scutellariae |
| MOL002932 | Panicolin | 76.25704989 | 0.2915 | Radix Scutellariae |
| MOL002933 | 5,7,4'-Trihydroxy-8-methoxyflavone | 36.56200469 | 0.26666 | Radix Scutellariae |
| MOL002934 | NEOBAICALEIN | 104.3446052 | 0.43917 | Radix Scutellariae |
| MOL002937 | DIHYDROOROXYLIN | 66.06173872 | 0.23057 | Radix Scutellariae |
| MOL008206 | Moslosooflavone | 44.08795959 | 0.25331 | Radix Scutellariae |
| MOL010415 | 11,13-Eicosadienoic acid, methyl ester | 39.27534422 | 0.2289 | Radix Scutellariae |
| MOL012245 | 5,7,4'-trihydroxy-6-methoxyflavanone | 36.62688628 | 0.26833 | Radix Scutellariae |
| MOL012246 | 5,7,4'-trihydroxy-8-methoxyflavanone | 74.23522001 | 0.26479 | Radix Scutellariae |
| MOL012266 | rivularin | 37.94023355 | 0.3663 | Radix Scutellariae |
| MOL002776 | Baicalin | 40.12 | 0.75 | Radix Scutellariae |
| MOL001918 | paeoniflorgenone | 87.59312084 | 0.36678 | Radix Paeoniae Alba |
| MOL001919 | (3S,5R,8R,9R,10S,14S)-3,17-dihydroxy-4,4,8,10,14-pentamethyl-2,3,5,6,7,9-hexahydro-1H-cyclopenta[a]phenanthrene-15,16-dione | 43.55620167 | 0.53276 | Radix Paeoniae Alba |
| MOL001924 | paeoniflorin | 53.87037516 | 0.78709 | Radix Paeoniae Alba |
