# Supplementary material for: Integrating Network Pharmacology and Metabolomics to Elucidate the Mechanism of Action of Huang Qin Decoction for Treament of Diabetic Liver Injury
Source: Front Pharmacol. 2022 May 25;13:899043. doi: 10.3389/fphar.2022.899043 (PMC9176298; doi:10.3389/fphar.2022.899043)
Supplement: Supplementary file 7 [file Table4.docx]

**Table 4 Information on the targets of Scutellaria baicalensis soup for DLD in network pharmacology**

| **No.** | Gene symbol | Protein name | Uniport ID |
| --- | --- | --- | --- |
| 1 | TGFB1 | Transforming growth factor beta-1 proprotein [Cleaved into: Latency-associated peptide | P01137 |
| 2 | SLC6A4 | Sodium-dependent serotonin transporter | P31645 |
| 3 | PTGS2 | Prostaglandin G/H synthase 2 | P35354 |
| 4 | PTGS1 | Prostaglandin G/H synthase 1 | P23219 |
| 5 | PRKCA | Protein kinase C alpha type | P17252 |
| 6 | PRKACA | cAMP-dependent protein kinase catalytic subunit alpha | P17612 |
| 7 | PON1 | Serum paraoxonase/arylesterase 1 | P27169 |
| 8 | PIK3CG | Phosphatidylinositol 4,5-bisphosphate 3-kinase catalytic subunit gamma isoform | P48736 |
| 9 | OPRM1 | Mu-type opioid receptor | P35372 |
| 10 | JUN | Transcription factor AP-1 | P05412 |
| 11 | HTR2A | 5-hydroxytryptamine receptor 2A | P28223 |
| 12 | HSP90AA1 | Heat shock protein HSP 90-alpha | P07900 |
| 13 | CASP9 | Caspase-9 | P55211 |
| 14 | CASP8 | Caspase-8 | Q14790 |
| 15 | CASP3 | Caspase-3 | P42574 |
| 16 | BCL2 | Apoptosis regulator Bcl-2 | P10415 |
| 17 | BAX | Apoptosis regulator BAX | Q07812 |
| 18 | ADRB2 | Beta-2 adrenergic receptor | P07550 |
| 19 | NR3C2 | Mineralocorticoid receptor | P08235 |
| 20 | XDH | Xanthine dehydrogenase/oxidase [Includes: Xanthine dehydrogenase | P47989 |
| 21 | VCAM1 | Vascular cell adhesion protein 1 | P19320 |
| 22 | TNF | Tumor necrosis factor | P01375 |
| 23 | STAT1 | Signal transducer and activator of transcription 1-alpha/beta | P42224 |
| 24 | SLPI | Antileukoproteinase | P03973 |
| 25 | SLC2A4 | Solute carrier family 2, facilitated glucose transporter member 4 | P14672 |
| 26 | SELE | E-selectin | P16581 |
| 27 | RELA | Transcription factor p65 | Q04206 |
| 28 | PRSS1 | Trypsin-1 | P07477 |
| 29 | PPP3CA | Serine/threonine-protein phosphatase 2B catalytic subunit alpha isoform | Q08209 |
| 30 | PPARG | Peroxisome proliferator-activated receptor gamma | P37231 |
| 31 | NR1I3 | Nuclear receptor subfamily 1 group I member 3 | Q14994 |
| 32 | NR1I2 | Nuclear receptor subfamily 1 group I member 2 | O75469 |
| 33 | NOS3 | Nitric oxide synthase, endothelial | P29474 |
| 34 | NOS2 | Nitric oxide synthase, inducible | P35228 |
| 35 | MMP1 | Interstitial collagenase | P03956 |
| 36 | MAPK8 | Mitogen-activated protein kinase 8 | P45983 |
| 37 | INSR | Insulin receptor | P06213 |
| 38 | IKBKB | Inhibitor of nuclear factor kappa-B kinase subunit beta | O14920 |
| 39 | ICAM1 | Intercellular adhesion molecule 1 | P05362 |
| 40 | HMOX1 | Heme oxygenase 1 | P09601 |
| 41 | GSTM1 | Glutathione S-transferase Mu 1 | P09488 |
| 42 | F7 | Coagulation factor VII | P08709 |
| 43 | F2 | Prothrombin | P00734 |
| 44 | DPP4 | Dipeptidyl peptidase 4 | P27487 |
| 45 | CYP3A4 | Cytochrome P450 3A4 | P08684 |
| 46 | CYP1A1 | Cytochrome P450 1A1 | P04798 |
| 47 | CDK1 | Cyclin-dependent kinase 1 | P06493 |
| 48 | AR | Androgen receptor | P10275 |
| 49 | ALOX5 | Polyunsaturated fatty acid 5-lipoxygenase | P09917 |
| 50 | AKT1 | RAC-alpha serine/threonine-protein kinase | P31749 |
| 51 | AHR | Aryl hydrocarbon receptor | P35869 |
| 52 | ACHE | Acetylcholinesterase | P22303 |
| 53 | RXRA | Retinoic acid receptor RXR-alpha | P19793 |
| 54 | ESR1 | Estrogen receptor | P03372 |
| 55 | LBP | Lipopolysaccharide-binding protein | P18428 |
| 56 | IL6 | Interleukin-6 | P05231 |
| 57 | CD14 | Monocyte differentiation antigen CD14 | P08571 |
| 58 | FASN | Fatty acid synthase | P49327 |
| 59 | TP53 | Cellular tumor antigen p53 | P04637 |
| 60 | THBD | Thrombomodulin | P07204 |
| 61 | SPP1 | Osteopontin | P10451 |
| 62 | SOD1 | Superoxide dismutase [Cu-Zn] | P00441 |
| 63 | SERPINE1 | Plasminogen activator inhibitor 1 | P05121 |
| 64 | RUNX2 | Runt-related transcription factor 2 | Q13950 |
| 65 | RB1 | Retinoblastoma-associated protein | P06400 |
| 66 | RAF1 | RAF proto-oncogene serine/threonine-protein kinase | P04049 |
| 67 | PTEN | Phosphatidylinositol 3,4,5-trisphosphate 3-phosphatase and dual-specificity protein phosphatase PTEN | P60484 |
| 68 | PRKCB | Protein kinase C beta type | P05771 |
| 69 | PPARD | Peroxisome proliferator-activated receptor delta | Q03181 |
| 70 | PPARA | Peroxisome proliferator-activated receptor alpha | Q07869 |
| 71 | PLAU | Urokinase-type plasminogen activator | P00749 |
| 72 | PLAT | Tissue-type plasminogen activator | P00750 |
| 73 | PARP1 | Poly [ADP-ribose] polymerase 1 | P09874 |
| 74 | ODC1 | Ornithine decarboxylase | P11926 |
| 75 | NQO1 | NAD | P15559 |
| 76 | NFKBIA | NF-kappa-B inhibitor alpha | P25963 |
| 77 | NFE2L2 | Nuclear factor erythroid 2-related factor 2 | Q16236 |
| 78 | MYC | Myc proto-oncogene protein | P01106 |
| 79 | MPO | Myeloperoxidase | P05164 |
| 80 | MMP9 | Matrix metalloproteinase-9 | P14780 |
| 81 | MMP3 | Stromelysin-1 | P08254 |
| 82 | MMP2 | 72 kDa type IV collagenase | P08253 |
| 83 | MGAM | Maltase-glucoamylase, intestinal [Includes: Maltase | O43451 |
| 84 | MAPK1 | Mitogen-activated protein kinase 1 | P28482 |
| 85 | IL2 | Interleukin-2 | P60568 |
| 86 | IL1B | Interleukin-1 beta | P01584 |
| 87 | IL1A | Interleukin-1 alpha | P01583 |
| 88 | IL10 | Interleukin-10 | P22301 |
| 89 | IGFBP3 | Insulin-like growth factor-binding protein 3 | P17936 |
| 90 | IGF2 | Insulin-like growth factor II | P01344 |
| 91 | IFNG | Interferon gamma | P01579 |
| 92 | HSPB1 | Heat shock protein beta-1 | P04792 |
| 93 | HSPA5 | Endoplasmic reticulum chaperone BiP | P11021 |
| 94 | HK2 | Hexokinase-2 | P52789 |
| 95 | HIF1A | Hypoxia-inducible factor 1-alpha | Q16665 |
| 96 | GJA1 | Gap junction alpha-1 protein | P17302 |
| 97 | FOS | Proto-oncogene c-Fos | P01100 |
| 98 | F3 | Tissue factor | P13726 |
| 99 | F10 | Coagulation factor X | P00742 |
| 100 | ERBB3 | Receptor tyrosine-protein kinase erbB-3 | P21860 |
| 101 | ERBB2 | Receptor tyrosine-protein kinase erbB-2 | P04626 |
| 102 | EGFR | Epidermal growth factor receptor | P00533 |
| 103 | E2F1 | Transcription factor E2F1 | Q01094 |
| 104 | CXCL8 | Interleukin-8 | P10145 |
| 105 | CXCL2 | C-X-C motif chemokine 2 | P19875 |
| 106 | CXCL10 | C-X-C motif chemokine 10 | P02778 |
| 107 | CTSD | Cathepsin D | P07339 |
| 108 | CRP | C-reactive protein [Cleaved into: C-reactive protein | P02741 |
| 109 | COL3A1 | Collagen alpha-1 | P02461 |
| 110 | CHEK2 | Serine/threonine-protein kinase Chk2 | O96017 |
| 111 | CDKN1A | Cyclin-dependent kinase inhibitor 1 | P38936 |
| 112 | CD40LG | CD40 ligand | P29965 |
| 113 | CCND1 | G1/S-specific cyclin-D1 | P24385 |
| 114 | CCL2 | C-C motif chemokine 2 | P13500 |
| 115 | CAV1 | Caveolin-1 | Q03135 |
| 116 | BIRC5 | Baculoviral IAP repeat-containing protein 5 | O15392 |
| 117 | BCL2L1 | Bcl-2-like protein 1 | Q07817 |
| 118 | AKR1B1 | Aldo-keto reductase family 1 member B1 | P15121 |
| 119 | ACACA | Acetyl-CoA carboxylase 1 | Q13085 |
| 120 | MAOA | Amine oxidase [flavin-containing] A | P21397 |
| 121 | ADRB1 | Beta-1 adrenergic receptor | P08588 |
| 122 | KDR | Vascular endothelial growth factor receptor 2 | P35968 |
| 123 | CTNNB1 | Catenin beta-1 | P35222 |
| 124 | CASP7 | Caspase-7 | P55210 |
| 125 | DRD2 | D | P14416 |
| 126 | CYP2B6 | Cytochrome P450 2B6 | P20813 |
| 127 | ESR2 | Estrogen receptor beta | Q92731 |
| 128 | CDK2 | Cyclin-dependent kinase 2 | P24941 |
| 129 | PTPN1 | Tyrosine-protein phosphatase non-receptor type 1 | P18031 |
| 130 | OLR1 | Oxidized low-density lipoprotein receptor 1 | P78380 |
| 131 | MAPK14 | Mitogen-activated protein kinase 14 | Q16539 |
| 132 | GSK3B | Glycogen synthase kinase-3 beta | P49841 |
| 133 | SIRT1 | NAD-dependent protein deacetylase sirtuin-1 | Q96EB6 |
| 134 | MT-ND6 | NADH-ubiquinone oxidoreductase chain 6 | P03923 |
| 135 | IL4 | Interleukin-4 | P05112 |
| 136 | STAT3 | Signal transducer and activator of transcription 3 | P40763 |
| 137 | CDK4 | Cyclin-dependent kinase 4 | P11802 |
| 138 | JAK2 | Tyrosine-protein kinase JAK2 | O60674 |
| 139 | SLC2A1 | Solute carrier family 2, facilitated glucose transporter member 1 | P11166 |
| 140 | MAPK10 | Mitogen-activated protein kinase 10 | P53779 |
| 141 | SREBF1 | Sterol regulatory element-binding protein 1 | P36956 |
| 142 | MTTP | Microsomal triglyceride transfer protein large subunit | P55157 |
| 143 | MAPK3 | Mitogen-activated protein kinase 3 | P27361 |
| 144 | LDLR | Low-density lipoprotein receptor | P01130 |
| 145 | HMGCR | 3-hydroxy-3-methylglutaryl-coenzyme A reductase | P04035 |
| 146 | GSR | Glutathione reductase, mitochondrial | P00390 |
| 147 | CYP19A1 | Aromatase | P11511 |
| 148 | CES1 | Liver carboxylesterase 1 | P23141 |
| 149 | BAD | Bcl2-associated agonist of cell death | Q92934 |
| 150 | APOB | Apolipoprotein B-100 | P04114 |
| 151 | ADIPOQ | Adiponectin | Q15848 |
| 152 | ABCC1 | Multidrug resistance-associated protein 1 | P33527 |
| 153 | CAT | Catalase | P04040 |
| 154 | UGT1A1 | UDP-glucuronosyltransferase 1A1 | P22309 |
| 155 | CYP2E1 | Cytochrome P450 2E1 | P05181 |
| 156 | FN1 | Fibronectin | P02751 |
| 157 | MCL1 | Induced myeloid leukemia cell differentiation protein Mcl-1 | Q07820 |
| 158 | PRKCD | Protein kinase C delta type | Q05655 |
| 159 | FASLG | Tumor necrosis factor ligand superfamily member 6 | P48023 |
| 160 | CYCS | Cytochrome c | P99999 |
| 161 | CYP2C9 | Cytochrome P450 2C9 | P11712 |
