# Supplementary material for: Integrating Network Pharmacology and Metabolomics to Elucidate the Mechanism of Action of Huang Qin Decoction for Treament of Diabetic Liver Injury
Source: Front Pharmacol. 2022 May 25;13:899043. doi: 10.3389/fphar.2022.899043 (PMC9176298; doi:10.3389/fphar.2022.899043)
Supplement: Supplementary file 8 [file Table6.docx]

**Table 6 Information on key metabolic pathways, metabolites and key targets**

| Related pathways | Key target genes | Key metabolites |
| --- | --- | --- |
| Glycerophospholipid metabolism | MAPK3、AKT1、MAPK8 | sphinganine，sphingosine |
| Tryptophan metabolism | PTGS2、CAT | Glutahione，Oxidized gutahione，Dihydrolipoamide |
